# Supplementary material for: Deletion of wfs1 Impairs Oligodendrocyte Precursor Cells Dorsal Distribution and Myelination Through the wfs1-hmgcs1 Axis in Zebrafish
Source: Biology (Basel). 2026 Aug 16;15(16):1402. doi: 10.3390/biology15161402 (PMC13510151; doi:10.3390/biology15161402)
Supplement: Supplementary file 1 [file biology-15-01402-s001.zip › supplementary material.pdf]

Supplementary Material

Deletion of *wfs1* Impairs Oligodendrocyte Precursor Cells Migration and Myelination Through the *wfs1*-*hmgcs1* Axis in Zebrafish

This file includes:

A. Supplementary table

Supplementary Table S1

B. Supplementary Figures

Supplementary Figures S1-S4

**Primer**	**Primer sequences (5' to 3')**	**Usage**
β-actin-F	CCCTGTTCCAGCCATCCTT	qPCR
β-actin-R	TTGAAAGTGGTCTCGTGGATACC	qPCR
mbp-F	CTGGGCAGAAAGAAGAAG	qPCR
mbp-R	GATGACCACGAAATGAAC	qPCR
mpz-F	TCACAGCAAAAACAGCGTATCT	qPCR
mpz-R	GCCGGCCTACAGGAAGTAAT	qPCR
plp1b-F	CAGGAATCACCCTTCTTG	qPCR
plp1b-R	GAACCTAGCAACGGATTC	qPCR
mag-F	GTCCAGCACCCTCACTTTCA	qPCR
mag-R	CAGTTCGAGGGGCGTATCTG	qPCR
wfs1b-F	GCTAAAGCACAGACAGAGATTGG	KO test
wfs1b-R	GTCCATTTTTGGCTGCCTGG	KO test
wfs1a-F	TGGTGTCCAATGAATCTAGCTCT	KO test
wfs1a-R	CACCAGAGGGGCTTAGTGTG	KO test
hmgcs1-F	CAGCTCTCCGAATCTAACCG	qPCR
hmgcs1-R	TCCAGCTCTGCTTGGTCAAC	qPCR
atf6-F	CTGTGGTGAAACCTCCACCT	qPCR
atf6-R	CATGGTGACCACAGGAGATG	qPCR
xbp1s-F	ACCAGTTAAGCTGGAAGCCC	qPCR
xbp1s-R	GTTTCAGAGAAGGCGACGGA	qPCR
hspa5-F	ACGAGAACACAGAAGACGGG	qPCR
hspa5-R	ATCCCAATCACTGTCCCAAC	qPCR
chop-F	GAGGACACGTAGAGAAGGGG	qPCR
chop-R	TCCGTTGAGCTCCACATTCTT	qPCR

Supplementary Table S1. Plasmid constructs primer sequences.

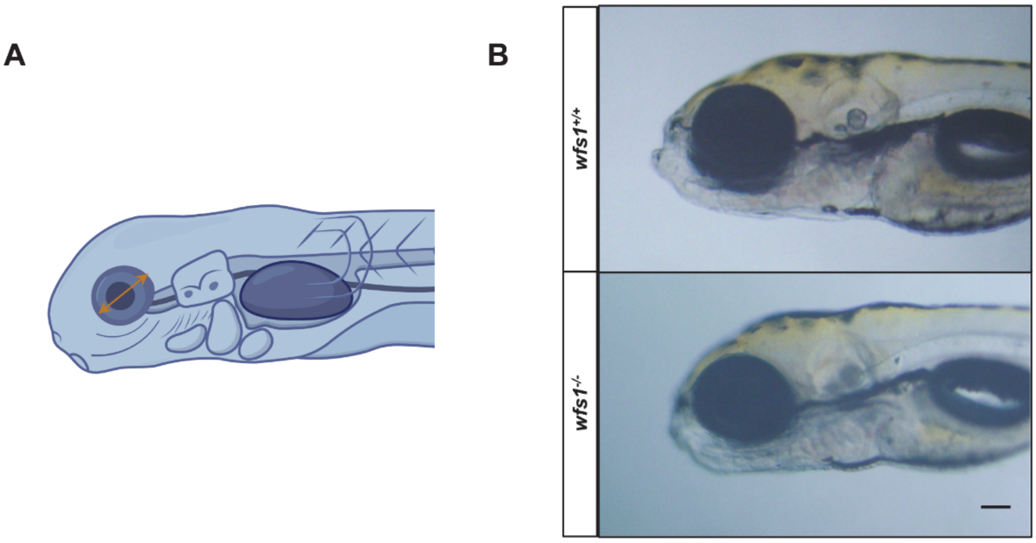

Supplementary Figure S1. Quantification of eye area in *wfs1*$^{-/-}$ zebrafish larvae. (A) Schematic illustration of the method used to quantify zebrafish eye area. The arrow indicates the measured eye diameter. (B) Representative bright-field microscopy images of the eye region in *wfs1*$^{+/+}$ and *wfs1*$^{-/-}$ larvae at 5 dpf. Scale bar: 100 µm.

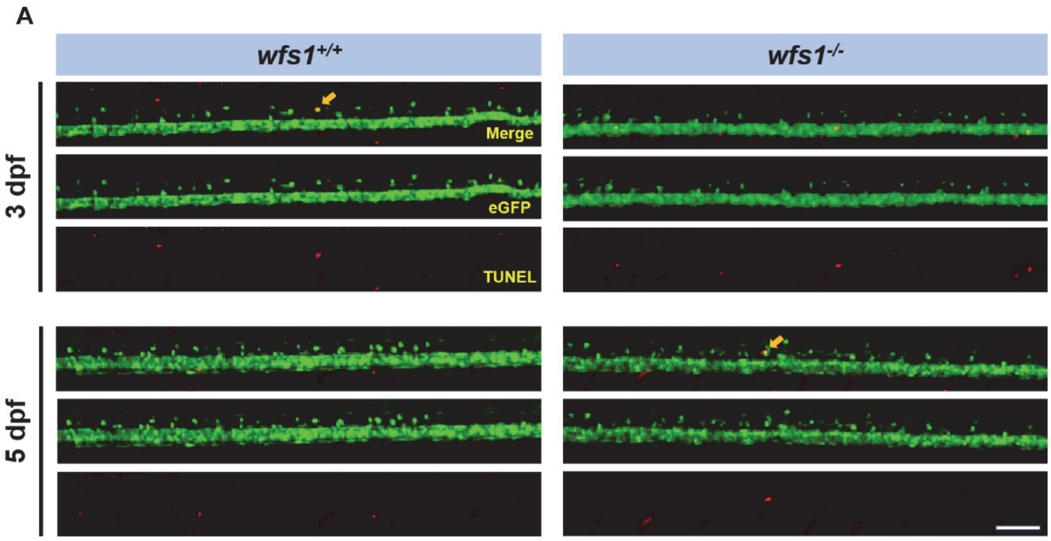

Supplementary Figure S2. OPCs rarely undergo apoptosis in *wfs1*$^{-/-}$ larvae at 3 dpf and 5 dpf. (A) Representative confocal images showing merged channels, *olig2*:EGFP fluorescence (green) and TUNEL staining (red) in *wfs1*$^{+/+}$ and *wfs1*$^{-/-}$ larvae at 3 dpf and 5 dpf. Arrows indicate TUNEL^{+}/EGFP^{+} double-positive cells. Almost no TUNEL^{+}/EGFP^{+} double-positive cells were detected within the quantified dorsal region in either genotype at either time point. n = 13 larvae per group. Scale bar: 20 µm.

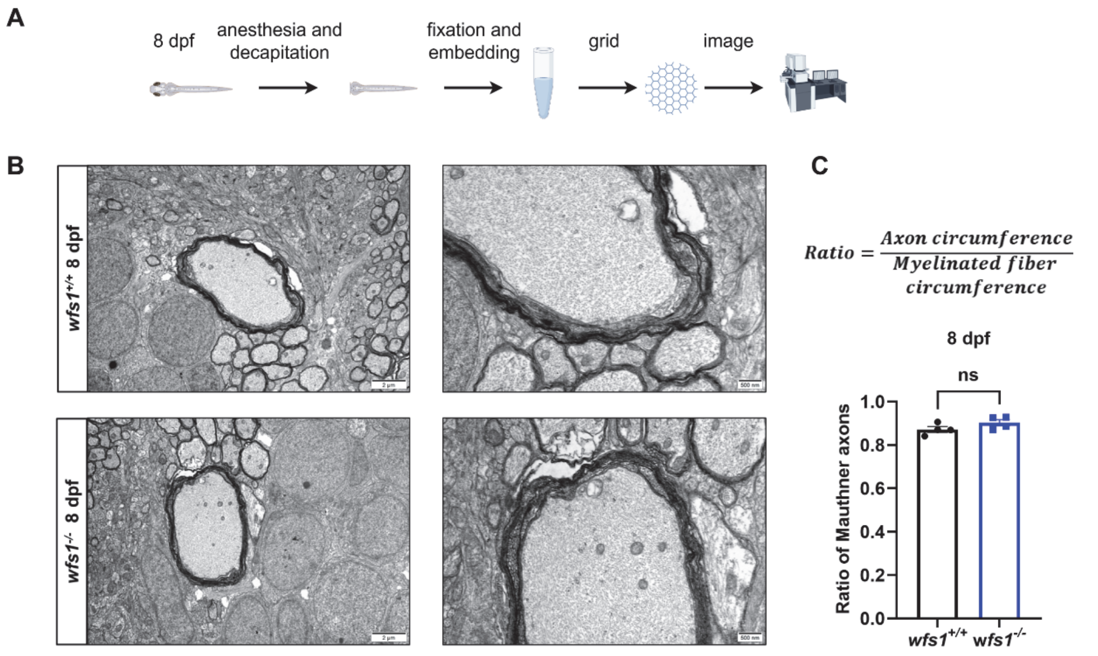

Supplementary Figure S3. The hypomyelination phenotype in *wfs1*$^{-/-}$ mutants emerges during early developmental stages and recovers by 8 dpf. (A) Experimental workflow for sample preparation and transmission electron microscopy observation of Mauthner cell axonal myelination in 8 dpf zebrafish larvae. (B) Transmission electron micrographs of the axons of Mauthner cells in *wfs1*$^{+/+}$ and *wfs1*$^{-/-}$ groups of 8 dpf zebrafish larvae. Scale bars: 2 µm (left), 500 nm (right). (C) Myelin thickness analysis of Mauthner cells in *wfs1*$^{+/+}$ and *wfs1*$^{-/-}$ groups. Data represent mean ± SEM and n = 4 biologically independent animals/group. ns, not significant, assessed by unpaired two-tailed Student's *t*-test.

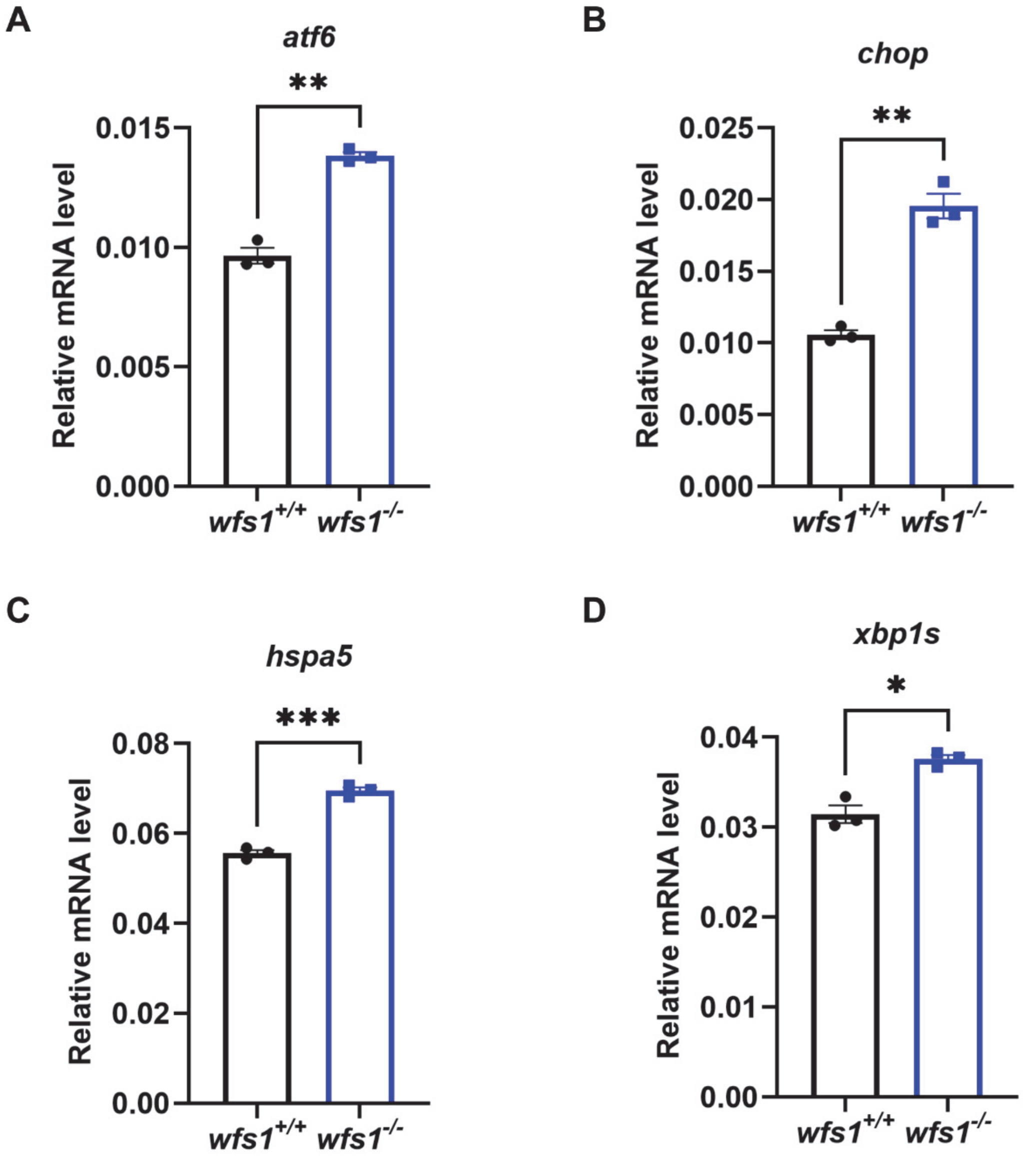

Supplementary Figure S4. ER stress markers are upregulated in *wfs1*$^{-/-}$ zebrafish at 5 dpf. (A-D) qRT-PCR analysis of canonical ER stress marker genes *atf6*, *chop*, *hspa5* (*bip*), and *xbp1s* in whole larvae at 5 dpf. Data represent mean ± SEM; n = 3 biologically independent replicates per group, each consisting of pooled RNA from 30 larvae. *$P < 0.05$, **$P < 0.01$, ***$P < 0.001$, assessed by unpaired two-tailed Student's *t*-test.
